# The Representation of Inflammatory Signals in the Brain – A Model for Subjective Fatigue in Multiple Sclerosis

**DOI:** 10.3389/fneur.2014.00264

**Published:** 2014-12-11

**Authors:** Katrin Hanken, Paul Eling, Helmut Hildebrandt

**Affiliations:** ^1^Department of Neurology, Klinikum Bremen-Ost, Bremen, Germany; ^2^Donders Institute for Brain, Cognition and Behaviour, Radboud University Nijmegen, Nijmegen, Netherlands

**Keywords:** multiple sclerosis, subjective fatigue, inflammation, pro-inflammatory cytokines, neuroimmunomodulation, insula, anterior cingulate cortex, hypothalamus

## Abstract

In multiple sclerosis (MS) patients, fatigue is rated as one of the most common and disabling symptoms. However, the pathophysiology underlying this fatigue is not yet clear. Several lines of evidence suggest that immunological factors, such as elevated levels of pro-inflammatory cytokines, may contribute to subjective fatigue in MS patients. Pro-inflammatory cytokines represent primary mediators of immune-to-brain-communication, modulating changes in the neurophysiology of the central nervous system. Recently, we proposed a model arguing that fatigue in MS patients is a subjective feeling, which is related to inflammation. Moreover, it implies that fatigue can be measured behaviorally only by applying specific cognitive tasks related to alertness and vigilance. In the present review, we focus on the subjective feeling of MS-related fatigue. We examine the hypothesis that the subjective feeling of MS-related fatigue may be a variant of inflammation-induced sickness behavior, resulting from cytokine-mediated activity changes within brain areas involved in interoception and homeostasis including the insula, the anterior cingulate, and the hypothalamus. We first present studies demonstrating a relationship between pro-inflammatory cytokines and subjective fatigue in healthy individuals, in people with inflammatory disorders, and particularly in MS patients. Subsequently, we discuss studies analyzing the impact of anti-inflammatory treatment on fatigue. In the next part of this review, we present studies on the transmission and neural representation of inflammatory signals, with a special focus on possible neural concomitants of inflammation-induced fatigue. We also present two of our studies on the relationship between local gray and white matter atrophy and fatigue in MS patients. Finally, we discuss some implications of our findings and future perspectives.

## Introduction

In multiple sclerosis (MS) patients, fatigue is rated as one of the most common and disabling symptoms. Its prevalence ranges from 65 to 97%, and it tends to seriously impair approximately one-third of all MS patients (1–4). Fatigue significantly impairs a patient’s quality of life, bearing negative effects on performance at work and on the patient’s social and private life (2, 5). Despite many investigations, the pathophysiology underlying MS-related fatigue is not yet clear. Proposed mechanisms for fatigue include primary causes such as gray matter atrophy (6–8), demyelination and axonal loss (9), functional cortical reorganization (10, 11), neuroendocrine dysregulation (12) as well as an immune system dysfunction (13, 14). On the other hand, also secondary causes such as sleep problems, medication, and depression have been suggested to be associated with MS-related fatigue (15, 16).

**Figure 1:**
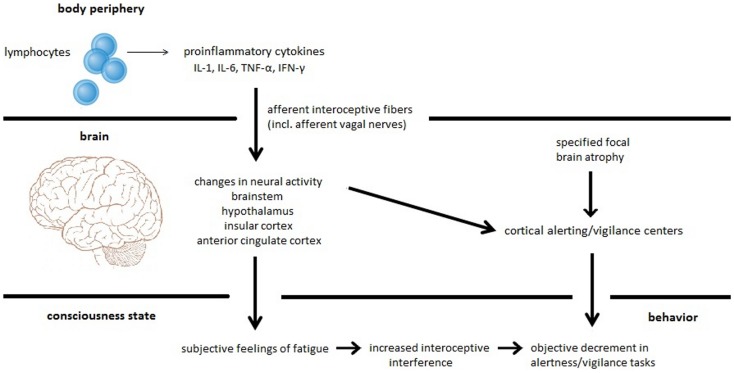
**Proposed model for MS-related fatigue**. Peripherally released pro-inflammatory cytokines IL-1, IL-6, TNF-α, and INF-γ activate immune-to-brain communication pathways such as afferent interoceptive nerve fibers (particularly afferents of the vagus nerve). These afferent nerve fibers innervate interoceptive and homeostatic brain areas including regions of the brainstem, the hypothalamus, the insula, and the anterior cingulate. Inflammation-induced activity changes within these brain regions cause the subjective feeling of fatigue. Furthermore, interoceptive information processing constitutes interoceptive interference resulting in a distraction of cognitive processes such as alertness and vigilance tasks that heavily rely on intrinsic alertness. This specific fatigue-related alertness and vigilance decrement can be exaggerated by focal brain atrophy affecting the alertness/vigilance network.

Based on our recently performed review on the relation between fatigue, cognitive performance, and brain atrophy in MS patients (17), we proposed a new model of MS-related fatigue. This model argues that subjective fatigue is a feeling resulting from inflammation-induced neural processing within interoceptive and homeostatic brain areas. Moreover, it argues that fatigue is only associated with specific cognitive states, such as alertness and vigilance, which depend on a high level of endogenous attention and which can be easily distracted by internal events like thoughts, feelings, and emotions (18). Hence, increased focusing on interoceptive aspects due to inflammation may disturb information processing of external stimuli and may interfere with sustained attention to a vigilance task causing a decrease in performance. Additionally, we suggest that this specific performance decrement may be exaggerated by brain atrophy or neurochemical dysfunction affecting the alerting/vigilance system (see Figure 1). Figure 1 (lower part) comprises the two different central phenomena, which we believe a complete theory of fatigue has to explain, i.e., subjective fatigue as a feeling and objective fatigue as the measurable decrement in behavioral performance. It also shows the two different causes (inflammation-induced changes in neural activity and specified focal brain atrophy), which can lead either to the feeling of fatigue and the objective impairment in sustained attention tasks or to the impairment in sustained attention tasks alone.

In the present review, we focus on the first aspect, namely the explanation of fatigue as a subjective feeling resulting from inflammation. Pro-inflammatory cytokines are elevated during inflammation and appear to represent primary mediators of the immune-to-brain-communication. Peripheral pro-inflammatory cytokines act specifically on brain regions involved in interoception and homeostasis to initiate physiological and behavioral changes such as fatigue (19). Consequently, we hypothesize that the subjective feeling of MS-related fatigue may be a variant of inflammation-induced sickness behavior, resulting from cytokine-mediated activity changes within brain areas involved in interoception and homeostasis including the insula, the anterior cingulate, and the hypothalamus. To evaluate this hypothesis, we will look at studies that investigated neural correlates of peripheral inflammation.

## Fatigue and Pro-Inflammatory Cytokines in Healthy Individuals

Assuming that subjective fatigue is a feeling resulting from central actions of increased peripheral pro-inflammatory cytokine concentrations, elevated levels of pro-inflammatory cytokines should also cause fatigue in healthy individuals. Weisdorf et al. (20) have demonstrated the important role of pro-inflammatory cytokines in the generation of fatigue by showing that several pro-inflammatory cytokines such as IL-1, IL-2, IL-6, IFN-γ, and TNF-α cause fatigue and somnolence in healthy individuals when administered exogeneously.

Furthermore, when healthy individuals become sick, they generate sickness behavior. The term sickness behavior has been used to refer to behavior that may be regarded as an adaptive response to acute infections or injuries (21). Everyone who has been suffering from a viral or bacterial infection will know what it means to “feel sick.” Major symptoms of sickness behavior may include fatigue, depression, anhedonia, anorexia, and increased pain sensitivity (22, 23). The syndrome may be fully present in some patients but only partly in others, depending on the severity and nature of inflammatory processes (24). The physiological and behavioral components of sickness behavior represent a highly organized strategy of an organism to cope with the infection. Sickness behavior appears to be primarily induced and regulated by pro-inflammatory cytokines such as IL-1α, IL-1β, TNF-α, and IL-6 (25–27). These cytokines are produced at the site of infection by activated immune cells and act centrally to induce physiological and behavioral components of sickness behavior (26, 28). This evidence suggests that the neurophysiology of sickness behavior may be responsible for the generation of inflammation-related fatigue.

Kerr et al. (27) studied healthy individuals, individuals at the time of acute viral infection (human parvovirus B 19) and after a mean follow-up period of 22.5 month. They demonstrated that circulating levels of TNF-α and IFN-γ were raised during acute and convalescent viral infection and that these elevated cytokine levels were strongly associated with subjective feelings of fatigue. Similar findings were reported by Hannestad et al. (29). These authors found that endotoxin-induced systemic inflammation increased serum levels of TNF-α and IL-6 as well as subjective fatigue in healthy individuals.

Kluge et al. (30, 31) analyzed immunomodulatory effects of antipsychotic drugs olanzapine and clozapine, which frequently produce sedation and sleepiness that share many similarities to fatigue (32). These researchers found that both drugs activate the cytokine system. Clozapine treatment was predominantly associated with an increase in TNF-α, sTNFr-1, sTNFr-2, IL-2r, and IL-6, whereas olanzapine treatment was found to be related to an increase in TNF-α, sIL-2r, and sTNFr-2.

## Fatigue and Pro-Inflammatory Cytokines in Inflammatory Disorders

Assuming that subjective fatigue is a feeling related to elevated pro-inflammatory cytokine levels, fatigue should be a major symptom in disorders with an underlying inflammatory pathophysiology. Actually, fatigue is a frequent complaint of patients suffering from inflammatory disorders such as chronic fatigue syndrome (33, 34), cancer (35), or autoimmune and autoinflammatory diseases such as systemic lupus erythematosus (36, 37), rheumatoid arthritis (38), Sjögren’s syndrome (39), and MS (3). Fatigue is also often reported by patients suffering from diseases that show signs of inflammation such as traumatic brain injury (40), stroke (41), Parkinson disease (42), sleep apnea (43), and human immunodeficiency virus infection (44). All these disorders are characterized by increased pro-inflammatory cytokine concentrations, strengthening the assumption that subjective fatigue may be due to elevated pro-inflammatory cytokines and their effect on the central nervous system (CNS).

Maes et al. (23) measured inflammatory markers in 107 patients with Myalgic Encephalomyelitis/Chronic Fatigue Syndrome (ME/CFS), 37 patients with chronic fatigue, and 20 healthy individuals. They found higher serum levels of IL-1 and TNF-α in patients with ME/CFS than in chronic fatigue patients and healthy controls. Furthermore, they found a significant correlation between increased serum IL-1, TNF-α, and subjective fatigue in patients suffering from ME/CFS. Meyers et al. (45) studied 54 patients with acute myelogenous leukemia and myelodysplastic syndrome before treatment initiation. They demonstrated significantly increased levels of circulating cytokines IL-1, IL-1ra, IL-6, IL-8, TNF-α, impaired cognitive functions, and elevated levels of subjective fatigue in these patients. Increased concentrations of IL-6, IL-1ra, and TNF-α were significantly correlated to subjective fatigue. Similar results were obtained by Bower et al. (46) who compared serum markers associated with pro-inflammatory cytokine activity in 20 fatigued breast cancer survivors and 20 non-fatigued survivors. Fatigued breast cancer survivors showed significantly higher serum levels of IL-1ra, sTNFr-2, and neopterin than survivors without fatigue. Moreover, cancer-related fatigue is commonly exacerbated by radio- and chemotherapy, which is thought to increase serum levels of pro-inflammatory cytokines (45, 47, 48). Greenberg et al. (49) examined this issue by evaluating the effect of radiotherapy on subjective fatigue and serum IL-1 in 15 men receiving radiation treatment for prostate cancer. They observed an association between the rise in serum IL-1 and the increase in subjective fatigue during radiotherapy. Cameron et al. (50) performed a longitudinal study (from time of treatment to 12 month later) investigating serum cytokine levels in 13 breast cancer patients with fatigue and 15 controls without post-cancer fatigue and did not find significant differences in cytokine levels between these two groups. However, the blood sampling for the analysis was conducted several weeks after the penultimate treatment cycle. Thus, relevant changes in cytokine concentration associated with treatment-related fatigue might have been missed. Moreover, the number of participants was very small and might have led to a Type II statistical error. Ormstad et al. (41) investigated the association between cytokine serum levels 72 h after stroke onset and fatigue scores at 6 and 12 month in 45 ischemic stroke patients. They found that acute serum levels of IL-1β positively correlated with the fatigue score at 6 month after stroke.

Most of these studies point to an association between pro-inflammatory cytokines such as IL-1, IL-6, and TNF-α and fatigue in disorders characterized by elevated cytokine levels. This well-documented association between elevated pro-inflammatory cytokines and increased subjective fatigue may well have implications for the explanation of fatigue. Thus, research on the relation between subjective fatigue and pro-inflammatory cytokines appears to be of great interest for a better understanding of MS-related fatigue.

## Fatigue and Pro-Inflammatory Cytokines in Multiple Sclerosis Patients

Multiple Sclerosis is considered to be an autoimmune inflammatory disorder of the CNS, in which autoreactive T-lymphocytes recognize CNS-specific proteins resulting in inflammation, demyelination, and axon degeneration (51). Pro- and anti-inflammatory cytokines are commonly up-regulated in parallel in most MS patients (52). Compared to healthy individuals, MS patients display increased serum and cerebrospinal fluid levels of pro-inflammatory cytokines such as IFN-γ, TNF-α, lymphotoxin-α, IL-2, IL-1β, and anti-inflammatory cytokines such as IL-10, IL-13, and TGF-β (52, 53). Given that pro-inflammatory cytokines have been linked to fatigue in various conditions with an underlying immunomodulatory pathology, it is not surprising that fatigue is regarded as one of the most common and disabling symptoms in MS (2–4).

Several lines of evidence suggest that immune factors play a major role in MS-related fatigue, supporting our hypothesis that MS-related fatigue might be some sort of inflammation-induced sickness behavior resulting from cytokine-induced changes in CNS neurophysiology. MS patients often complain of a higher fatigue level during relapses, which are characterized by an increased immune activation, representing an up-modulation of pro-inflammatory cytokines such as TNF-α, IL-1, IL-6, and lymphotoxin-α (52, 54–56). Moreover, the administration of immunomodulatory medication such as interferon-beta frequently causes short-term effects such as reversible fatigue in MS (55, 57, 58). Goebel et al. (59) studied the effect of interferon-beta (IFN-β-1b) on plasma levels of inflammatory cytokines in eight healthy men. They found that interferon-beta injection led to an immediate increase in TNF-α, IL-6, and IL-10 plasma levels. Nicoletti et al. (60) studied the impact of short-term interferon-beta treatment on blood cytokine levels in 14 relapsing-remitting MS patients. They found that MS patients treated with interferon-beta showed increased serum levels of IL-6, IFN-γ, and IL-10.

Studies on the relationship between pro-inflammatory cytokines and MS-related fatigue demonstrated a significant association between subjective fatigue and the stimulated production capacity for IFN-γ and TNF-α (14, 61). Pokryszko-Dragan et al. (61) evaluated the stimulated production of IFN-γ by peripheral CD3^+^- and CD4^+^-T lymphocytes in 20 MS patients with and 20 without fatigue as determined by the Fatigue Severity Scale (FSS). They found an increased stimulated IFN-γ production in severely fatigued MS patients. Heesen et al. (14) compared whole blood stimulatory capacity for pro- (TNF-α, IFN-γ) and anti-inflammatory (IL-10) cytokines in 15 MS patients with and 15 MS patients without fatigue as determined by the FSS. They found that patients with fatigue displayed significantly increased TNF-α and IFN-γ production capacities. Flachenecker et al. (13) reported similar findings by studying 37 MS patients. They demonstrated a significant association between TNF-α mRNA expression in peripheral blood cells and FSS scores, independent from age, disease duration, disease course, disability, interferon treatment, or signs of autonomic dysfunction. Finally, Bertolone et al. (62) measured serum levels of IL-1β, Il-6, β-2-microglobolin, sIL-2r, and soluble CD8 in 30 MS patients with severe fatigue. They found a significant correlation between beneficial effects of amantadine and pemoline on subjective fatigue and reductions in serum levels of IL-1β and IL-6.

On the other hand, Rudick and Barna (63) did not find significant differences in IL-2 levels comparing 8 fatigued MS patients and 50 healthy controls. Other studies that failed to demonstrate an association between inflammatory processes and MS-related subjective fatigue did not measure direct pro-inflammatory cytokine concentrations (64, 65). Instead, they analyzed concentrations of inflammatory markers such as urinary neopterin or they measured indirect effects of pro-inflammatory cytokines.

Summing up, studies on the relationship between pro-inflammatory cytokines and MS-related subjective fatigue highlight an association between elevated pro-inflammatory cytokines IFN-γ, TNF-α, IL-1, IL-6, and increased feelings of fatigue. These findings support our hypothesis that subjective fatigue in MS patients might be a variant of inflammation-induced sickness behavior resulting from cytokine-induced changes in CNS neurophysiology.

## Anti-Inflammatory Treatment and Fatigue

Providing that pro-inflammatory cytokines and their effect on the CNS induce the feeling of fatigue, anti-inflammatory treatment should reduce subjective fatigue. Actually, anti-TNF-α treatment strategies have shown to ameliorate subjective fatigue in patients suffering from rheumatoid arthritis and sleep apnea (66, 67). Anakinra, an IL-1 receptor antagonist used in rheumatoid arthritis, also showed significant improvements on fatigue scores (68). In patients with Sjögren’s syndrome, inhibition of IL-1β caused a 50% reduction in subjective fatigue (39). Finally, bupropion, a psychopharmacological drug with anti-inflammatory properties against TNF-α, has shown to reduce excessive daytime sleepiness (69).

If subjective fatigue in MS patients represents an internal state resulting from increased pro-inflammatory cytokine levels, anti-inflammatory treatment should also have beneficial effects on fatigue in MS patients. However, there are hardly any studies on the effect of anti-inflammatory cytokines on MS-related fatigue. Glatiramer acetate, used in the treatment of MS, has anti-inflammatory properties and seems to reduce fatigue in MS patients (70, 71). Furthermore, natalizumab treatment, which was found to reduce circulating plasma levels of TNF-α, IL-6, and IL-10 as well as cerebrospinal fluid levels of IL-1β, IL-6, and IL-8, seems to have a beneficial effect on subjective fatigue in MS patients (72–75). Interestingly, aerobic exercise leads to a reduction in subjective fatigue in MS patients (76, 77). Regular aerobic exercise in MS patients was found to induce anti-inflammatory actions such as the stimulated production of anti-inflammatory cytokines and the inhibited production of pro-inflammatory cytokines TNF-α and IFN-γ (78). Therefore, the beneficial effect of aerobic exercise on MS-related fatigue may be due to its anti-inflammatory implications. Finally, body cooling, which was found to have a positive impact on MS-related fatigue, also seems to decrease pro-inflammatory cytokine (IL-1) production by peripheral blood cells (79, 80).

All these observations point to a beneficial effect of anti-inflammatory treatment options on subjective fatigue in disorders with elevated levels of pro-inflammatory cytokines, supporting our hypothesis of a relationship between pro-inflammatory cytokines and fatigue.

## Neuronal Aspects of Fatigue – Transmission and Representation of Inflammatory Signals in the Brain

If subjective fatigue is a feeling such as anxiety or pain, one would expect this feeling to be represented cortically. Consequently, the question arises which brain areas are associated with processing the feeling fatigue. According to our hypothesis, we expect brain areas, related to central effects of peripheral inflammation and immunomodulation, to be associated with fatigue. To understand how peripheral pro-inflammatory cytokines may produce this feeling of fatigue, we will now review studies on the transmission and central representation of peripheral inflammatory signals and its association with fatigue.

It is commonly presumed that peripherally released cytokines act on the brain via two pathways: one fast neural transmission pathway involving primary afferent nerves innervating the body site of inflammation and a slow humoral transmission pathway involving cytokines originating from the choroid plexus and circumventricular organs (22, 28, 81).

Studies have shown that primary afferent nerves, especially afferents of the vagus nerve, play a key role in the neural transmission of peripheral immune signals to the brain (82–86). For example, immunohistochemical studies demonstrated an activation of vagal primary afferent neurons after having treated rats with peripheral endotoxin or IL-1β (87, 88). Other animal studies have shown that sectioning the abdominal vagus nerve abolished most brain-mediated illness responses induced by the peripheral administration of endotoxin or IL-1β (89, 90). Sensory neurons of the vagal nerve appear to possess receptors for pro-inflammatory cytokines and the activation of afferent nerve fibers by peripherally released cytokines presumably represents a fast pathway and direct activation of specified brain targets (26, 81). However, the specific neural substrates that process immunosensory information remain elusive. Animal experiments using immunohistochemistry to detect the expression of c-Fos identified immunoreactive neurons in the primary projection area of the afferent vagus nerves, represented by the nucleus tractus solitaris, and in secondary projection areas such as the parabrachial nucleus, the hypothalamic paraventricular and supraoptic nuclei, the thalamus, the bed nucleus of the stria terminalis, the central nucleus of the amygdala, the insular cortex, the anterior cingulate cortex (ACC), and the medial prefrontal cortex (91–95). All these brain structures are implicated in homeostasis and in the representation of internal bodily states (interoception). However, only few animal experiments analyzed the association between central and behavioral effects of pro-inflammatory cytokines. Gaykema et al. (90, 91) studied the effect of lipopolysaccharide challenge on behavior and neural activity (Fos expression) in rats. Shortly after systemic peripheral inflammation, rats presented symptoms of sickness behavior, such as fatigue. Furthermore, researchers demonstrated a significant relation between symptoms of sickness behavior and suppressed activity of orexinergic and histaminergic neurons located in the hypothalamus.

In humans, a growing number of neuroimaging studies have investigated central effects of peripheral inflammation and have generally confirmed the important role of the insula and the ACC in immunomodulation (29, 96–99). Rosenkranz et al. (99) used functional magnetic resonance imaging (fMRI) to study the role of the CNS in the regulation of inflammation in allergic asthmatic patients. They found an association between peripheral TNF-α in response to immunological challenge and activity in the ACC as well as between eosinophils and activity in the insula. Similar results were obtained by Eisenberger et al. (97) who analyzed the relationship between neural activity using fMRI and pro-inflammatory cytokine activity in individuals exposed to endotoxin. The authors found an association between endotoxin-induced elevations in IL-6 and increased neural activity in the dorsal ACC and the anterior insula in females but not in males. Ohira et al. (98) recorded immune indices and regional cerebral blood flow in men, using positron emission tomography. They observed a correlation between the increase in natural killer cells and the increase in regional cerebral blood flow in the left insula, the medial and bilateral orbitofrontal cortex, and in the anterior middle prefrontal cortex. Furthermore, they demonstrated a correlation between a decrease in T helper cells and a decrease in regional cerebral blood flow in the right insula and the medial orbitofrontal cortex. Hannestad et al. (29) analyzed this issue by using positron emission tomography to identify brain regions that are involved in the response to endotoxin administration in humans. This research group found that systemic inflammation causes an increase in peripheral TNF-α and IL-6 concentrations. Moreover, they found that endotoxin administration led to a higher normalized glucose metabolism in the insula and to a lower normalized glucose metabolism in the ACC. Summing up, nearly all of these studies demonstrated a relationship between inflammatory markers and activity changes within the insula and the ACC. Only one neuroimaging study, performed by Harrison et al. (96), examined the relationship between inflammation-induced activity changes within the brain and inflammation-induced fatigue. These authors demonstrated that systemic inflammation in healthy individuals causes an increase in neural activity in the insula and the anterior cingulate and that these activity changes predict variations in inflammation-associated fatigue. Moreover, the authors showed that the association between inflammation-associated fatigue and increased activity in the insula and anterior cingulate relies on afferent, rather than on efferent autonomic effects, suggesting that fatigue as a core symptom of sickness behavior emerges from afferent interocepive information processing.

These findings point to an implication of interoceptive and homeostatic brain regions like the insula, the anterior cingulate, and the hypothalamus in immunomodulation and suggest that these areas might represent neural correlates of inflammation-induced fatigue.

## The Possible Role of the Insula, the Anterior Cingulate and the Hypothalamus in the Generation of Inflammation-Related Subjective Fatigue

Assuming that subjective fatigue is a feeling represented in cortical areas that are involved in interoception and homeostasis, we now take a closer look at the brain regions that have been found frequently to be implicated in inflammation: the insula, the ACC, and the hypothalamus.

In human beings, convergent afferent vagal and spinal interoceptive fibers terminate in the anterior insula providing a central representation of well-being. Craig et al. (100) found that activity in the posterior insula correlated with stimulus intensities, whereas activity in the anterior insula correlated with subjective feelings of these stimuli intensities, suggesting that the anterior insula provides a basis for the generation of subjective feelings. The insula and the ACC have both been implicated in sensing and responding to physiological disturbances (101). Some authors suggest that afferent homeostatic signaling is integrated in the anterior insula and that the subsequent efferent response is driven by the ACC (101, 102). According to that hypothesis, inflammation would activate all regions of the insula resulting in the generation of subjective feelings of sickness behavior such as fatigue. On the other hand, the ACC would provide the basis for ongoing adjustments to behavior and physiology to restore and maintain our well-being.

The hypothalamus was found to be related to inflammation-induced fatigue in animal experiments and is an important structure for regulating wakefulness and sleep. Orexinergic neurons in the lateral hypothalamus and histaminergic neurons located in the posterior hypothalamus play a key role in inducing and maintaining wakefulness and vigilance (103). Consequentially, observed inflammation-driven inhibition of orexinergic and histaminergic neurons in the hypothalamus might contribute to subjective fatigue as well as to fatigue-related vigilance impairment.

## Studies on the Involvement of the Insula, the Anterior Cingulate and the Hypothalamus in MS-Related Fatigue

Zellini et al. (104) used T1 relaxation time as a sensitive measure to indicate pathological changes in the hypothalamus in 44 relapsing-remitting MS patients. Compared to 13 healthy controls, MS patients had a significantly higher T1 relaxation time in the hypothalamus. Moreover, the authors found a significant positive correlation between T1 relaxation times and patients’ fatigue scores, as assessed with the FSS. These findings point to an association between pathological changes in the hypothalamus and MS-related fatigue, supporting our hypothesis that the hypothalamus, especially histaminergic and orexinergic neurons, might play an important role for fatigue in MS patients.

Recently, we investigated the association between the integrity of posterior hypothalamic fibers and the level of cognitive fatigue in 49 relapsing-remitting MS patients using diffusion tensor imaging (105). We found that non-cognitively fatigued patients revealed greater axial and radial diffusivity for fibers between brainstem areas and the posterior hypothalamus, indicating tissue loss. This tissue loss might have resulted from demyelination and/or degeneration of investigated fibers including afferent interoceptive fibers and afferents of the vagal nerve that innervate the posterior hypothalamus, including the histaminergic system and other brain regions such as the insular cortex. Consequently, loss of fiber integrity might reduce inflammation-induced suppression of histaminergic neurons as well as inflammation-induced activity in the insula, resulting in a decreased feeling of fatigue.

In another recent study, we analyzed the association between subjective fatigue and cortical thickness in two independent data sets, encompassing in total 96 relapsing-remitting MS patients (106). In both data sets, regression analysis revealed thickness of the right insular cortex as an independent predictor of the patients’ FSS score. Patients without fatigue had a thinner right insular cortex than patients with fatigue, suggesting that the right insular cortex plays an important role in the generation of fatigue and that atrophy in this area apparently results in a decrease of fatigue.

Hesse et al. (107) used positron emission tomography and a serotonin transporter-selective tracer to investigate serotonergic activity in 23 MS patients and 22 healthy controls. Compared to healthy controls, MS patients had lower serotonin transporter availability in the cingulate cortex, the thalamus, and the insula and increased availability in the orbitofrontal cortex. Moreover, the authors found a positive correlation between patients’ serotonin transporter availability in the insula and fatigue scores (assessed via the Würzburger Erschöpfungsinventar bei MS), pointing to an involvement of the insular cortex in the generation of MS-related fatigue.

Several lines of evidence suggest that atrophy as well as functional changes in the ACC are related to fatigue in MS patients (6, 8, 10, 108, 109). Multiple structural imaging studies found an association between increased white and gray matter atrophy in the ACC and subjective fatigue in MS patients (6, 8, 108). Furthermore, functional imaging studies found that MS patients with fatigue have a larger and more significant activation of the ACC during the execution of simple motor tasks than patients without fatigue (10, 109). These findings support our assumption that the ACC is an important neural structure related to MS-related fatigue.

## Implications of These Findings for Our Fatigue Model

We recently proposed a fatigue model arguing that two independent mechanisms may contribute to subjective and objective fatigue in MS patients: (1) subjective fatigue as a feeling is related to inflammation-induced information processing within interoceptive and homeostatic brain areas and (2) objective fatigue as the measureable decrement in behavioral performance is related to atrophy in the cortico-subcortical vigilance network [(17); see Figure 1].

We propose that subjective fatigue in MS patients is a feeling that reflects an internal state depending on interoceptive information processing. Thus, similar to pain, fatigue may contribute to increased interoceptive information processing and it may act as a source of interoceptive interference. Hence, our model proposes that subjective fatigue can be measured behaviorally only by applying specific cognitive tasks that rely on a high degree of *intrinsic alertness* such as vigilance and alertness tasks. Moreover, it argues that a vigilance and alertness decrement may be enhanced by brain atrophy and/or neurochemical dysfunction of the alerting/vigilance system. According to this model, fatigue in MS patients may differ depending on the disease progress. During disease onset inflammatory processes might predominantly cause subjective fatigue, whereas in later disease stages advanced brain atrophy of specified brain regions might predominantly contribute to objective fatigue. This assumption has implications for the treatment of MS-related fatigue: while anti-inflammatory treatment options might show beneficial effects during disease onset, it may not help any more in advanced disease stages.

In this review we focused on the association between inflammation, the subjective feeling of fatigue and its possible neural correlates. The empirical findings discussed above all point to a relationship between elevated levels of peripheral TNF-α, IFN-γ, IL-1β, and IL-6 and subjective fatigue, supporting our hypothesis that subjective fatigue in MS patients is related to inflammation. Furthermore, the findings demonstrate that elevated levels of peripheral pro-inflammatory cytokines activate afferent interoceptive fibers, including afferents of the vagus nerve which innervate brain regions involved in interoception and homeostasis, such as the insula (particularly the anterior insula), the anterior cingulate and the hypothalamus. Hence, we suggest that inflammation-induced activity changes in these brain regions may reflect the neural substrates of the feeling of fatigue.

In general, our fatigue model currently can best be tested by using vigilance and alertness tasks. Furthermore, MRI techniques like diffusion tensor imaging may be helpful in analyzing afferent nerve fibers that transmit inflammatory signals to the brain. Analysis of the relationship between cortical thickness or localized lesions in interoceptive brain regions and fatigue might support our fatigue model. To show that fatigue is a feeling related to inflammation that is represented in interoceptive/homeostatic brain regions like the insula, the ACC and the hypothalamus, functional imaging studies combined with the assessment of subjective fatigue and the evaluation of cytokine levels would be necessary.

## Conflict of Interest Statement

The authors declare that the research was conducted in the absence of any commercial or financial relationships that could be construed as a potential conflict of interest.
